# Perinatal SSRI exposure affects brain functional activity associated with whisker stimulation in adolescent and adult rats

**DOI:** 10.1038/s41598-021-81327-z

**Published:** 2021-01-18

**Authors:** Noortje Van der Knaap, Dirk Wiedermann, Dirk Schubert, Mathias Hoehn, Judith R. Homberg

**Affiliations:** 1 Donders Institute for Brain, Cognition and Behaviour, Radboud University and Radboud University Medical Center, 6500 HB Nijmegen, The Netherlands; 2 In-Vivo-NMR Laboratory, Max Planck Institute for Metabolism Research, Cologne, Germany; 3 Department of Cognitive Neuroscience, Donders Institute for Brain, Cognition and Behaviour, Radboud University Nijmegen Medical Centre, Kapittelweg 29, 6525 EN Nijmegen, The Netherlands

**Keywords:** Development of the nervous system, Somatosensory system

## Abstract

Selective serotonin reuptake inhibitors (SSRI), such as fluoxetine, are used as first-line antidepressant medication during pregnancy. Since SSRIs cross the placenta the unborn child is exposed to the maternal SSRI medication, resulting in, amongst others, increased risk for autism in offspring. This likely results from developmental changes in brain function. Studies employing rats lacking the serotonin transporter have shown that elevations in serotonin levels particularly affect the development of the whisker related part of the primary somatosensory (barrel) cortex. Therefore, we hypothesized that serotonin level disturbances during development alter brain activity related to whisker stimulation. We treated female dams with fluoxetine or vehicle from gestational day 11 onwards for 21 days. We investigated offspring’s brain activity during whisker stimulation using functional magnetic resonance imaging (fMRI) at adolescence and adulthood. Our results indicate that adolescent offspring displayed increased activity in hippocampal subareas and the mammillary body in the thalamus. Adult offspring exhibited increased functional activation of areas associated with (higher) sensory processing and memory such as the hippocampus, perirhinal and entorhinal cortex, retrospinal granular cortex, piriform cortex and secondary visual cortex. Our data imply that perinatal SSRI exposure leads to complex alterations in brain networks involved in sensory perception and processing.

## Introduction

According to the World Health Organization, depression is the leading worldwide cause of disability and affects individuals across all life stages, including pregnancy^1^. Fourteen to sixteen percent of the female population in The Netherlands reported depressive feelings during their child-bearing years^2^. The first-line treatment for depression involves selective serotonin reuptake inhibitors (SSRI), because of their minimal side-effects^3^. Of all Dutch pregnant women, 1.8–2% continue antidepressant use during pregnancy and 0.5% start using antidepressants during pregnancy^4^. While the SSRI treatment is directed at the mother, by passing the placenta the SSRIs can also reach the unborn child: SSRI levels have been measured in amniotic fluid and breast milk^5,6^. There is evidence that this has consequences for the developing child, as new-borns exhibit withdrawal symptoms after birth^7^, called Postnatal Adaption Syndrome, due to a sudden decline in SSRI exposure after birth. Furthermore, SSRI exposure during pregnancy has been linked to increased risk for pulmonary hypertension, low birth weight, congenital heart defects and defects in motor behaviour^8–12^. Additionally, studies focusing on the long-term effects of SSRI exposure on offspring found associations between prenatal SSRI exposure and increased behavioural problems, such as higher risk for Autism Spectrum Disorder^9,13,14^.

SSRIs block the serotonin transporter (SERT) that re-uptakes serotonin after it has been released by the synapse, lengthening serotonin’s presence in the extracellular space and prolonging its signal^3^. Serotonin is also an important regulatory peptide during development with functions in almost the entire brain, including the regulation of synaptogenesis, neuronal outgrowth and migration^15^. Therefore, different levels of prenatal serotonin can affect the brains’ development. Long-term controlled studies in humans are challenging because of the ethical difficulties of randomized controlled studies. Rodent models fill this gap, giving us the opportunity to closely investigate the consequences of developmental SSRI exposure. These preclinical studies have suggested differences in social behaviour, learning and memory, anxiety and sensory functions, alterations in neuronal organization, differences in hippocampal neurogenesis and epigenetic changes after SSRI exposure during pregnancy and the early postnatal phase^16–28^.

Several studies in rodents have demonstrated that elevated serotonin levels during brain development lead to prominent alterations in the organisation of the somatosensory system, in particular in the thalamocortical innervation and intracortical networks of the primary somatosensory (barrel) cortex. Perinatal SSRI exposure has been associated with reduced interhemispheric barrel cortex connectivity^29^. In rodents lacking SERT (SERT knockout) the barrel cortex is characterized by changes in the size and width of the barrels, as well as reduced inhibitory control over excitatory neurons and thereby probably altered sensory gating^30–33^. Furthermore, SERT knockout rats were found to be faster in sensory integration in the gap crossing task in which the animals use their whiskers to cross a gap between two platforms^30^. Further, cerebral glucose utilization was found to be reduced in the whisker-to-barrel cortex pathway of SERT knockout mice compared to control mice upon unilateral whisker stimulation^34^. Since there are similarities in the developmental consequences of SERT knockout and perinatal SSRI exposure^35,36^, and perinatal SSRI exposure affects sensory functions as well as behaviours dependent on sensory processing (see above), we hypothesize that perinatal SSRI exposure affects the activity of brain areas associated with the somatosensory system later in life.

To test this hypothesis, we sought to investigate the effect of perinatal SSRI (fluoxetine) exposure on the later life brain-wide response to whisker stimulation, using functional magnetic resonance imaging (fMRI). We have chosen this approach to maximize the translational findings to humans. More specifically, we treated dams from gestational day 11 until postnatal day 11 with fluoxetine. We have chosen this period as serotonergic neurons emerge at embryonic day 10–12 in rodents^37^, and postnatal day 2 to 11 constitutes a 5-HT-sensitive period impacting (sensory-dependent) anxiety-like behaviour in offspring^38^. Additionally, as postnatal day 10–11 in the rat corresponds to birth in humans^39^, with this treatment period we model SSRI exposure approximately during the second and third trimester of human pregnancy. In the offspring we studied how this perinatal SSRI exposure affected brain activity patterns associated with whisker stimulation at adolescence and adulthood.

## Material and methods

### Animals

**Figure 1:**
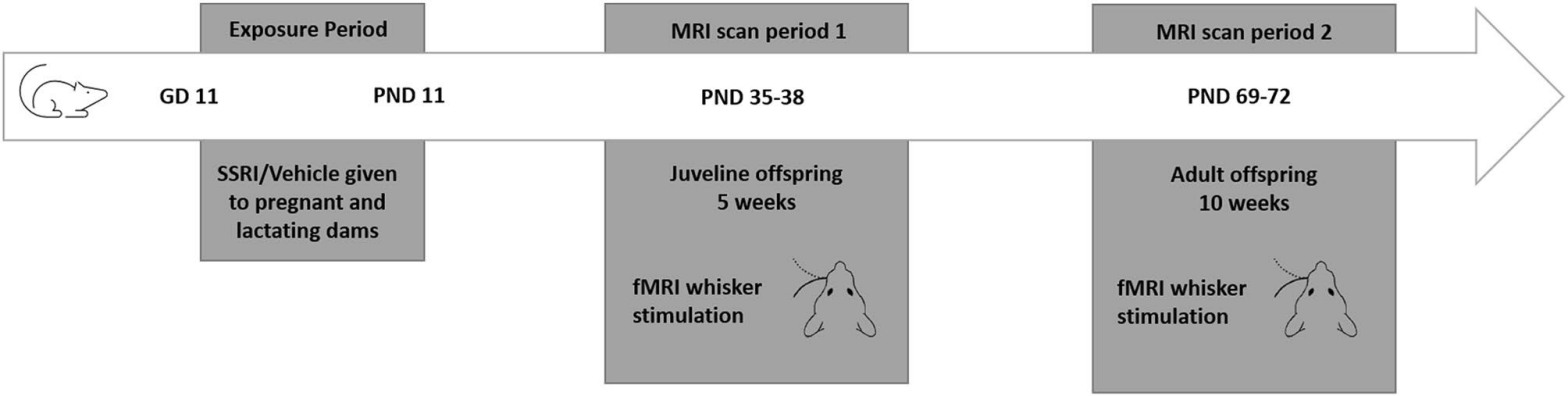
Model of the experimental timeline and set-up. *GD* gestational days, *PND* postnatal days.

Twelve female and five male Wistar rats (Harlan-Winkelmann GmbH, Borchen, Germany, and Janvier, Le Genest Saint Isle, Cedex, France) were housed under controlled temperature conditions (21  ± 1 °C), humidity (55%  ± 10%) and light/dark cycle (12/12 h) at the Max Planck Institute of Metabolism Research, Cologne. They were given access to food and water ad libitum. After 2 weeks of acclimation female rats were measured daily for their oestrous stage with an impedance checker (Impedance Checker MK-10B, Muromachi Kikai, Tokyo, Japan). When the females were in oestrous, they were paired with a male and placed in a cage with a custom-made wire bottom (stainless steel, containing small circular holes of 10 mm, spacing 13 mm). Twelve hours later male and female were separated and the cage was inspected for the vaginal plug. If found, the day was marked as gestational day (GD) 1. The female dams were handled daily and treated during the exposure period, via an oral gavage from GD 11 onwards for 21 days with 12 mg/kg fluoxetine or 1% methylcellulose (vehicle). In total 6 dams were treated with fluoxetine and 6 dams with vehicle. Pups derived from these dams were weaned at 3 weeks and inspected for sex. Male pups proceeded to the MRI procedures which were in total 26 animals for the control group and 15 for the SSRI group. All animals underwent two MRI periods (functional and structural) on two days at 5 and 10 weeks, representing adolescence and adulthood respectively (Fig. 1). Due to the long acquisition times and the need of different anaesthetics, the functional and the structural scans could not be performed in the same scanning session. Consequently, on day one a dedicated fMRI session was conducted, and on day two an anatomical scan was made. Three animals of the fluoxetine group and one animal in the control group were lost during MRI scans. All animal experiments were carried out in accordance with the guidelines of the German Animal Welfare Act and approved by the ethical committee for animal studies of the local authorities (Landesamt für Natur, Umwelt und Verbraucherschutz Nordrhein-Westfalen).

### Magnetic resonance imaging

MRI experiments were conducted on a 9.4 T horizontal bore dedicated animal scanner (Biospec 94/20, Bruker Biospin, Ettlingen, Germany). Radiofrequency transmission was achieved with a quadrature volume resonator (inner diameter 72 mm), and a rat brain surface coil (~ 30 × 30 mm^2^) (Bruker, Biospin) was used for signal detection. A custom-made cradle was used (Medres, Cologne, Germany) to perform the fMRI whisker stimulation. The head was fixated using a bite bar and ear bars to reduce movement artefacts. Respiration, heart rate and body temperature were controlled at all times during scanning. Body temperature was maintained at 37 °C via a controlled water circulation system integrated in the cradle (Medres, Cologne, Germany). Imaging was conducted with the Paravision software, version 5.1 (Bruker BioSpin).

The animals were carefully positioned to make sure that the barrel cortex was aligned with the isocenter of the magnet. To optimize field homogeneity, the implemented MAPSHIM of Paravision was used for shimming locally for the barrel cortex. For fMRI a gradient echo EPI sequence was used. TR/TE_eff_ = 3,502 ms/17.5 ms; field of view 28.8 mm × 28.8 mm (matrix 96 × 96) and 1.2 mm slice thickness; pixel size = 0.3 × 0.3 mm; 11 slices.

The T2-weighted imaging (T2WI) was acquired using a Multi Slice Multi (Spin-) Echo sequence (MSME; TR/TE = 5500 ms/10 ms; 16 echoes, slice thickness = 0.5 mm; 30 slices; in-plane resolution = 0.145 × 0.145 mm^2^. For the generation for anatomical images for registration within the fMRI procedure we used the TurboRARE sequence with a TR/TE_eff_ = 6500 ms/32.5 ms; field of view 32 mm × 32 mm (matrix 256 × 256) and 0.5 mm slice thickness; voxel size = 0.3 × 0.3 mm^2^; 11 slices.

### MRI scanning procedure

The MRI protocol contained a 2-day procedure and was conducted at 5 and 10 weeks of age. On day one the fMRI protocol was run. The animals were anesthetized with 2% isoflurane (Forane, Baxter, Deerfield, IL, USA) in a 70/30 mixture of N_2_0 and O_2_ and placed within the MRI cradle_._ The whiskers were prepped for fMRI whisker stimulation where, unilaterally, the whisker B1, C1, D1 and E1 were flagged together with tape while the other whiskers were carefully fixated away from the airflow. A tube with N_2_ was fixed to hit the flag in the middle and was tested with each animal for correct positioning before placing the animals within the scanner. The N_2_O was then replaced by N_2_. The animal was given a 0.5 ml bolus of 0.05 mg/kg of medetomidine (Domitor, Pfizer, Karlsruhe, Germany; 1 ml/kg in 10 ml of saline) subcutaneously^40^. Isoflurane was slowly discontinued and sedation was maintained via continuous subcutaneous infusion of a 1 ml/h methomidine solution (0.1 mg/kg). The fMRI procedure was conducted approximately 30 min after bolus injection. The TurboRARE was recorded for anatomical realignment of the fMRI image before the fMRI procedure was executed. The whiskers were stimulated with a N_2_ puff in an anterior to posterior deflection with a frequency of 6 Hz and a block design consisting of 5 blocks of activation of 15 s each intertwined with 45 s of no stimulation. The long periods of no stimulation were included to make sure that the somatosensory system would not desensitize due to overstimulation. After the imaging session, atipamezol (Anitsedan, Pfizer, 1 ml/kg bodyweight) was injected subcutaneously, together with 2 ml of saline, to reverse the sedative effect and overcome any fluid loss due to the diuretic effect of medetomidine solution. Twenty-four hours later animals were placed in the scanner again, this time under isoflurane anaesthetic without any whisker preparation for structural T2 weighted scans.

### Functional MRI analysis

Successful scans for the fMRI analysis were obtained from 14 control animals and 12 perinatally SSRI exposed animals at 5 weeks of age, and 18 control animals and 11 perinatally SSRI exposed animals at 10 weeks of age. These animals were used for further image analyses.

The fMRI images were converted to NIfTI format and voxel size in the header was scaled with a factor 10 to account for the brain size differences between rat and humans^41^. FSL version 4.0.5^42^ was used for all analysis unless stated otherwise. Images were pre-processed, with rigid body motion correction and temporal high pass filtering. Recorded breathing, cardiac and movement information obtained was used to regress out non-specific Blood Oxygen Level Dependent (BOLD) activation. fMRI images were aligned to its own structural image. The structural images were used to align to the in-house rat template image by means of ANTS^43,44^. Statistical analysis where performed in two steps with FEAT (fMRI expert analysis tool of FSL version 6.0). At the first level statistics, the statistical activation maps were calculated using general linear model (GLM) for the block design of whisker stimulation using the convolution of double-gamma Hemodynamic Response Function. GLM uses FILM (FMRIB’s improved linear model) to pre-whiten each voxel time series, to create robust estimation time-series (described in more detail elsewhere^45^). To compare the SSRI and vehicle treated groups at 5 and 10 weeks, we preformed FEAT’s second level comparative statistic with FLAME (FMRIB's Local Analysis of Mixed Effects) within FEAT, upon the statistical actions maps from first level statistics to compare differences in activation patterns. FLAME used Marko Chain Monte Carlo sampling to get an accurate estimation of the true random-effects variance and degrees of freedom at each voxel (as described in more detail elsewhere^46^). Upon the z-level statistical images, a mask of the whole brain in-house rat template (as described above) was used to exclude any voxels outside of the brain in the comparison, and a cluster level corrected statistic was performed with a z threshold of 2.4 and a cluster P threshold of < 0.05^47^.

### Voxel based morphometry analysis

Successful scans for the T2 weighted scans were obtained at 5 weeks of age from 15 control and 10 perinatally SSRI exposed animals, and at 10 weeks of age from 17 control and 11 perinatally SSRI exposed animals.

Like for the fMRI images, the T2 weighted images were converted to NIFTI format and resized with a factor of 10 to account the differences between human and rodent brain size. The procedures were further executed with SPM 12 (Welcome Trust Centre for Neuroimaging, London, UK). The mean of the T2 weighted images were realigned and then rigid body aligned to the high resolution Wistar atlas image^48^ and resized to 1.25 mm isotropic voxel. The image was then segmented (warping regularization: 1; warp frequency cut-off: 25; bias regularization: 0.0001; bias FWHM: 60 mm cut-off; and sampling distance: 3)) to GM (gray matter), WM (white matter) and CSF (cerebrospinal fluid) using the rat atlas tissue priors^48^. To obtain the most accurate registration of the segmented images, DARTEL (Diffeomorphic Anatomical Registration Through Exponentiated Lie algebra) was used for an automated, unbiased, and nonlinear template building^49^. After the DARTEL template building procedure the images were normalized either non-modulated or modulated to its own DARTEL age specific template and smoothed with an 8 × 8 × 8 mm FWHM Gaussian kernel. Both modulation approaches were used to inspect for volume and density differences between groups. A general linear model was conducted to compare the differences of treatment for each age group. We corrected for whole brain volume as global measurement. The images were thresholded with an exact mask of 0.2 to investigate the contrasts of control > perinatal SSRI exposure and perinatal SSRI > control exposure with a family wise error correction of p < 0.05.

## Results

### Functional MRI

**Figure 2:**
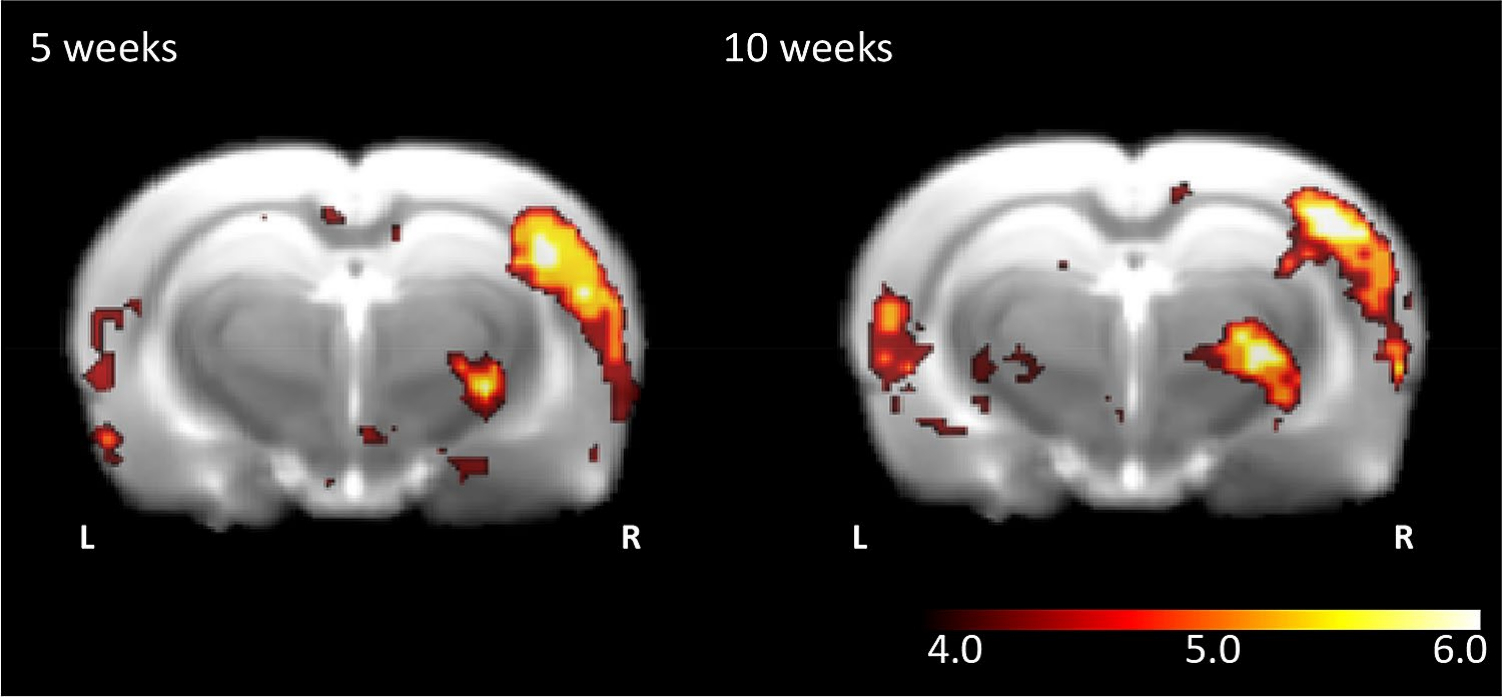
The effect of whisker stimulation block > control block over all subject groups. In both the perinatally SSRI exposed and control groups whisker stimulation to the left side is associated with increased activation of the barrel cortex at the contralateral side (5 weeks: z-max = 6.43, p < 0.001; 10 weeks: z-max is 6.25; p < 0.001). Additionally, there is strong activation in thalamic projection sides and sensory relay regions like the ventral posteromedial nucleus and posteromedial complex region. Cluster activation is shown on an in-house rat template and z-scores are depicted on the scale bar.

Generally, unilateral stimulation of one row of whiskers led to a most prominent and strong activation in the contralateral barrel field of the rat (Fig. 2). The stimulus induced activation pattern was revealed by means of the overall contrast of the whisker stimulation block over the control block and at both 5 (n = 26) and 10 (n = 29) weeks of age we observed large significant clusters of activation after correction for multiple comparisons (5 weeks: z-max = 6.43, p < 0.001; 10 weeks: z-max is 6.25; p < 0.001). However, fMRI also showed that the stimulation and resulting movement of the whiskers not only activated the barrel cortex. In 5-week-old rats, we found the peak maxima of the activity also in somatosensory nuclei of the thalamus, i.e. the thalamic ventral posteromedial nucleus (VPM) and posterior Thalamic (Po) group^50,51^. Furthermore, the stimulation procedure resulted in the activation of non-somatosensory areas, in particular in the primary auditory area. At 10 weeks of age the peak maxima were located in similar areas as at 5 weeks of age, including the barrel cortex, the VPM and Po region of the thalamus and in the primary auditory area. However, unlike at the younger age, whisker stimulation also led to peak maxima in medial secondary visual cortex, parietal association area, and perirhinal and entorhinal cortex (Table 1).

**Table 1:** Peak Maxima of overall contrast whiskers vs no whiskers stimulation p < 0.001.

	z-value	Area
5 weeks	6.43	Barrel cortex
	6.23	Barrel cortex
	5.96	Barrel Cortex
	5.91	Barrel cortex
	5.91	Ventral posteromedial thalamic nucleus and Posterior Thalamic group
	5.86	Secondary auditory cortex
10 weeks	6.25	Medial secondary visual cortex
	6.12	Associative parietal cortex
	6.11	Barrel cortex
	6.10	Primary auditory cortex, ectorhinal and perirhinal cortex
	5.84	Primary Auditory Cortex, Ectorhinal And Perirhinal cortex
	5.84	Ventral posteromedial thalamic nucleus and Posterior Thalamic group

**Figure 3:**
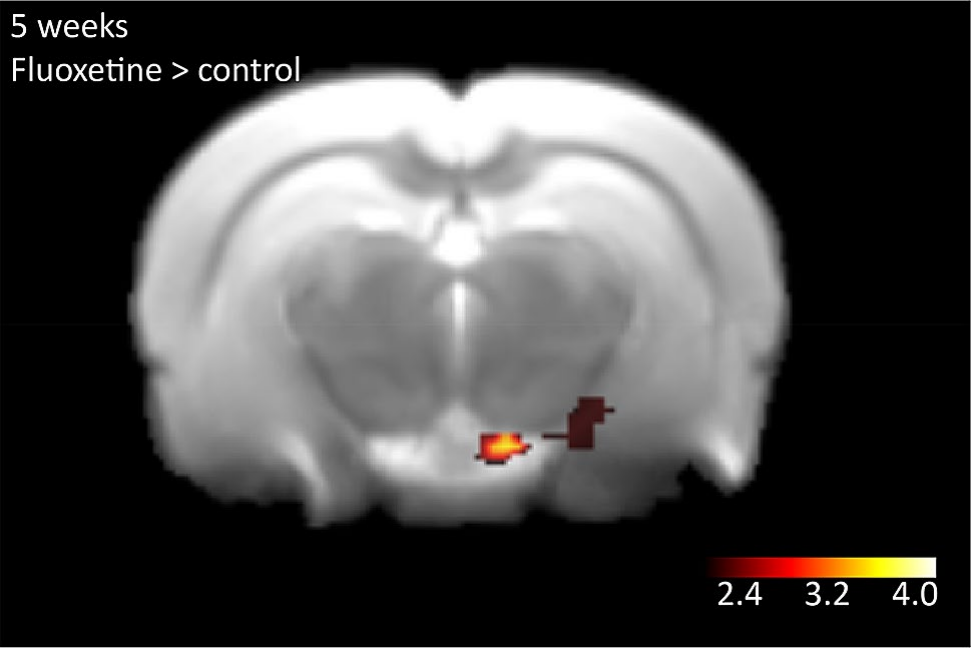
Results of the contrast perinatal SSRI exposure > control for the 5 weeks old rats. Whisker stimulation was given on the left side of the animal. Increased activity is observed in the hippocampal CA3 and dentate gyrus regions stretching to the mammillary body in the thalamus (cluster size = 72 voxels, z-max 1.9 p = 0.012). Significant clusters are shown upon an in-house rat template and z-scores are depicted in the scale-bar.

When comparing whisker stimulation evoked activity in brains of perinatal SSRI exposed animals at 5 weeks of age over the respective controls (perinatal SSRI exposure > control), we found no significant differences in barrel field activation. However, we did find differences in the CA3 and dentate gyrus subareas of the hippocampus, stretching to the mammillary body in the thalamus (cluster size = 72 voxels, z-max 1.9, p = 0.012) (Fig. 3). We found no differences for the contrast control > perinatal SSRI exposure.

**Figure 4:**
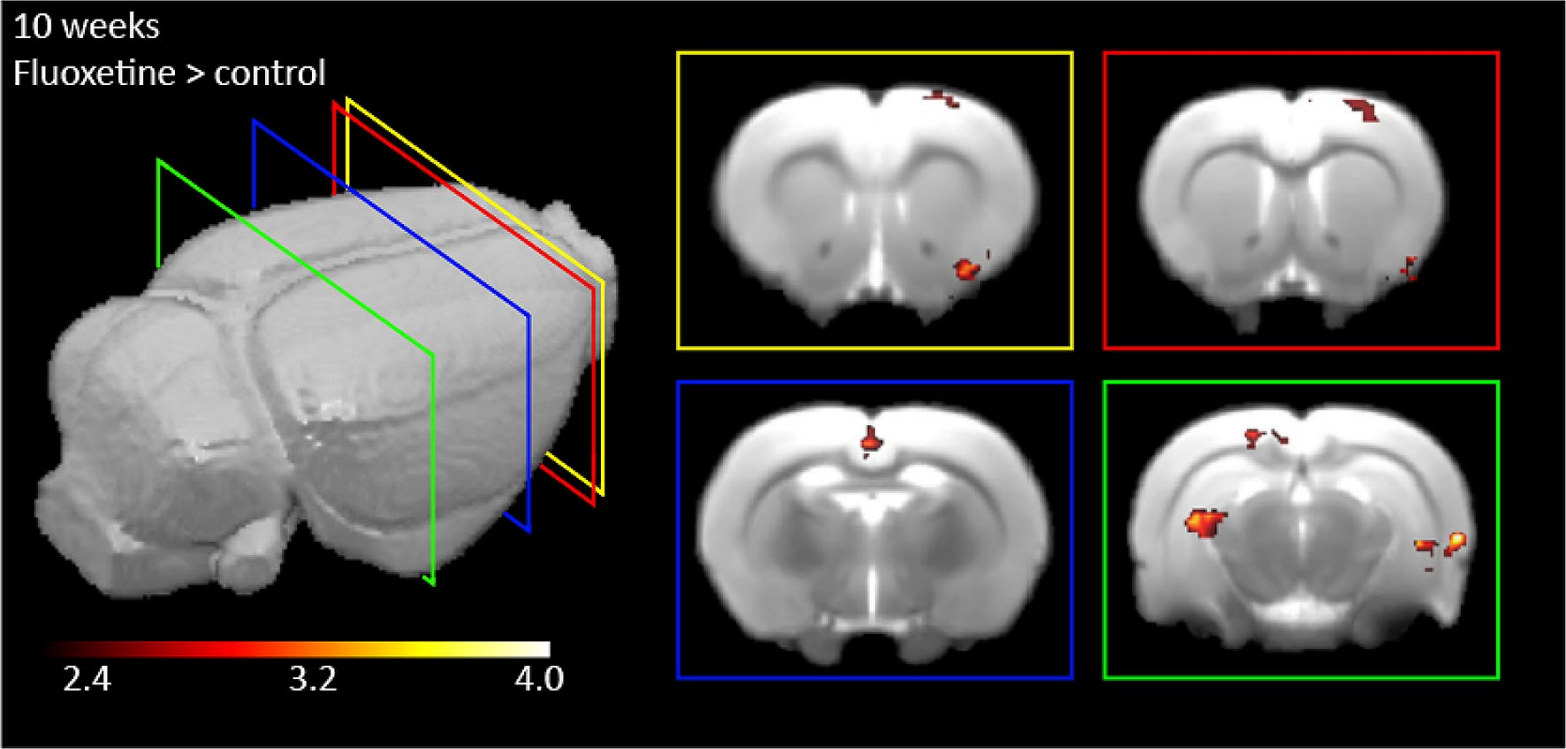
Results of the activated brain clusters in the 10-week old rats. The contrast for perinatal SSRI exposure over the control condition is shown. Whisker stimulation was given on the left side of the animal. Six different significant clusters are shown. The yellow square shows the contralateral activation of the piriform cortex (cluster size = 54 voxels, z-max = 3.81, p = 0.003). In both the yellow and red squares, the activation of the cluster encompassing the motor cortex and primary sensory cortex is shown (cluster size = 38 voxels, z-max = 3.0, p = 0.02). In the blue square the activation of the cluster in the retrosplenal granular and agranular cortex is shown (cluster size = 38 voxels, z-max = 3.2, p = 0.03). In the green square the activation of three clusters is shown: Ipsilateral to the stimulation side and most dorsal the secondary visual cortex (cluster size = 35 voxels, z-max = 3.63 p = 0.04), and the CA2 of the hippocampus (cluster size = 87 voxels, z-max = 4.93 and p < 0.001). Lastly, a cluster covering the perirhinal and entorhinal cortex stretching into the hippocampal CA2 region is activated (cluster size = 60 voxels, z-max = 4.07, p = 0.002). All clusters shown survived whole brain cluster correction and are laid over an in-house rat template. Value-bar depicts the z-value range.

Differences in barrel field activation were also not detectable between SSRI exposed brains and controls at 10 weeks of age. As in the 5-week-old animals, we did observe an increased signal in the perinatally SSRI exposed group over the control group in several other brain regions. However, the regions were to a large extent different from those found at 5 weeks of age (Fig. 4). Six clusters of activation survived whole-brain cluster-correction. As a whole, these detected regions are known to be involved in sensory information processing, sensory integrating, and memory. Most anterior we found increased activation of the piriform cortex (cluster size = 54 voxels z-max = 3.81, p = 0.003). Posterior to this and overlapping in location we observed activation in an area that was spanning from motor cortex (M1) to the primary somatosensory cortex (S1) (cluster size = 38 voxels, z-max = 3.0, p = 0.02). Around the Bregma location of AP − 2.5 we observed activation in the retrospinal (a)granular cortex (cluster size = 38 voxels, z-max = 3.2, p = 0.03). Most posterior (Bregma AP = − 5.0) we found three clusters. One cluster in the left CA2 hippocampal region (cluster size = 87 voxels, z-max = 4.93, p < 0.001), one cluster that is located in the perirhinal cortex and entorhinal region stretching into the right hippocampal CA2 region (cluster size = 60 voxels, z-max = 4.07, p = 0.002), and lastly an activation cluster in the secondary visual cortex (cluster size = 35 voxels, z-max = 3.63 p = 0.04). There is no cluster significant in the contrast control > perinatal SSRI exposure.

### Voxel based morphometry (VBM)

At 5 and 10 weeks of age no significant effects (p > 0.05) were found between the SSRI treated group and the control group for either volume or density differences in the voxel-based morphometry analysis.

## Discussion

In this study we set out to test how perinatal exposure to the SSRI fluoxetine affects large-scale functional brain activity associated with tactile sensory activation. We measured in adolescent and adult rats functional brain activity using fMRI during whisker stimulation, to obtain insight in the later life consequences of perinatal SSRI exposure. Rodents show large scale activation of the barrel cortex upon whisker stimulation, but no differences in the BOLD response in the barrel field were found between the control and perinatally SSRI exposed groups. However, in several regions involved in higher-level processing of sensory information or memory integration we observed increased activation in the perinatally SSRI exposed group compared to the control group, particularly in adult rats.

The long-term effects of prenatal SSRI exposure on human offspring have been shown to involve increased behavioural problems in later life, including a higher risk for Autism Spectrum Disorders^9,13,14^, which are characterized by altered sensory integration and function^52–54^. However, this association was not always replicated^55–57^. It has been reported that perinatal SSRI exposure in humans affects the function of the auditory cortex. These findings may relate to serotonin’s role in the regulation of sensory processes^58,59^, and the association between serotonergic genetic mutations and autism-related phenotypes as observed in animals^60^. This link of serotonin with sensory processing alterations and outcome is supported by our findings that early alterations in serotonin signalling due to SSRI exposure affect the integration of sensory information. This integration could on the long-term affect behaviour related to the autistic spectrum.

When inspecting our results, we did not observe in the barrel cortex itself significant differences in overall activation between exposure groups, although we specifically evoked whisker-related sensory input. It is possible that the limited spatial and time resolution of fMRI rendered small changes in the barrel cortex undetectable. It is also possible that over time, during development, changes in barrel cortex function due to perinatal SSRI exposure dissipated or restored but led to down-stream changes in other brain regions. The capability of the sensory cortical areas to compensate for structural distortions was shown in mouse models with severely distorted laminar organisation of the cortex^61^. Even in the absence of any normal structural correlate to barrels, imaging of whisker stimulation related activity revealed reinstated topological network recruitment^62^. A developmental normalization of barrel cortex function could explain why the general BOLD response in the barrel cortex is similar between groups at adolescent and adult age. Potentially, the plastic brain rewires when the serotonin levels are gradually normalized after postnatal day 11, when SSRI exposure has stopped. However, SSRI exposure still results in differential functional activation associated with sensory stimulation, but reflected in other brain regions.

When comparing the activation of whisker stimulation as main effect, adolescent and adult rodents showed a different pattern of peak maxima. The peak maxima in the adolescent group are mainly located in the barrel cortex, while the adults show activation in other sensory regions, like visual and auditory areas (Table 1)^63^. In the different exposure groups, perinatal SSRI over control, the adolescent group displayed activity differences in the hippocampus and mammillary body. In the adult rats, perinatal fluoxetine exposure led to more BOLD activity compared to vehicle exposure in response to whisker stimulation in other sensory regions than the barrel cortex. We observed activation in the piriform cortex which is involved in olfaction. In addition, we observed activation in the motor cortex and primary somatosensory cortex, secondary visual cortex and hippocampus. Moreover, we detected increased activation in the retrospinal granular cortex, involved in spatial judgement, the perirhinal cortex involved in sensory information processing, and the enthorinal cortex, involved in spatial mapping (Fig. 4). All these areas are implicated in the integration of environmental sensory information. These results were not due to differences in brain size, as shown by the VBM analysis.

The different activation patterns at adolescence and adulthood for the main task effect may be indicative of more integration of sensory input across development. Multisensory integration develops over age in humans, mostly before adulthood^64–66^. Multi-sensory integration is more pronounced at adult ages than during childhood, and used more often by adults than by children^67^. Our findings support this developmental view. Our results are also in line with studies in which tactile information was disrupted by removing whiskers during neonatal period and multi-sensory integration differences were found well into adulthood^68^. This pattern of results indicates that sensory (integration) systems are highly dynamic during development, able to overcome distortions to a certain extent. However, they always have to recalibrate within the limits of wiring and experience. Whether the increased activity in the different sensory processing regions as we found in the present study is a sign of a still developing sensory integration process or an indication that the brain is differentially calibrated due to the developmental setback after fluoxetine exposure, is unknown. Nonetheless, the present data provide initial insight into the brain-wide effects of the maternal use of SSRI on sensory processing.

Previous research revealed that rodents prenatally exposed to an SSRI exhibited decreased spatial memory performance^22^. Our results suggest that mostly hippocampal and memory-related regions show increased activation in perinatally fluoxetine exposed offspring compared to controls. This could be due to an implicit increase in memory integration or a result of increased activity in different sensory regions important for the integration of information and subsequent memory encoding.

This work has some limitations. Due to strict regulations in animal testing and the explorative nature of this experiment, we investigated a restricted number of animals in this study. Nonetheless, the present study shows that fMRI is a valid tool to investigate large-scale brain functional and structural changes due to developmental perturbations in serotonin levels. Another limitation is that the female rats treated with SSRIs were healthy, while in humans SSRIs are only provided to women having depressive symptoms. We therefore cannot draw conclusions regarding the potential adverse effects of maternal depression on brain-wide activity patterns in response to sensory stimulation. Further, while the dams were handled very gently before treatment, we cannot rule out the possibility that the oral gavage stressed the dams, which in turn could influence offspring brain development. Also, as we only tested the SSRI fluoxetine, it remains unknown whether findings also generalize to other SSRIs. Finally, we cannot exclude the possibility that the blue square in Fig. 4 presents the *venus sagitalis* rather than the retrosplenal and agranular cortex, as the fMRI signal in this position has previously been suggested to stem from this vene^69^. We would also like to point out that the anaesthesia we needed to apply to the animals during the imaging may have had effects on the stimulus evoked brain activity. However, based on previous reports^70^ we can expect that the resulting brain activity patterns closely reflected those of awake animals and that the anaesthesia was not a major confounding factor for detecting differences in the responses of untreated and SSRI exposed offspring.

We never experience our environment with one sensory system only. The integration of visual, tactile, vestibular, proprioceptive, auditory and olfactory information is how we structure the world and our place in it. The integration of sensory input and the integration of this input with memory processes is key to our fitness and known to develop well into adulthood^64^. Exposure to serotonin altering medication during development has been related to differences in neuronal and behavioural levels^16–28^. In this study we found brain-wide differences in functional activity during adolescence and adulthood as a consequence of perinatal SSRI exposure. Our findings suggest that developmental SSRI exposure affects sensory processing of the brain even into adulthood. Hence, future research should not focus only on primary sensory areas, but also examine the overarching picture of sensory integration in the entire brain after developmental disrupting influences, like antidepressant medication.
